# Expressed mutated genes in Sezary syndrome and their potential prognostic value in patients treated with extracorporeal photopheresis

**DOI:** 10.3389/fimmu.2025.1589467

**Published:** 2025-08-22

**Authors:** Cristina Cristofoletti, Giulia Salvatore, Cristian Bassi, Massimo Negrini, Giovanni Luca Scaglione, Luca Mazzarella, Gianmaria Frigè, Ylenia Aura Minafò, Martina Fioretti, Alessandro Monopoli, Maria Pina Accetturi, Maria Antonietta Pilla, Cosimo Di Raimondo, Alessandra Frezzolini, Enrico Scala, Stefania D’Atri, Giandomenico Russo, Maria Grazia Narducci

**Affiliations:** ^1^ Laboratory of Molecular Oncology, Istituto Dermopatico dell'Immacolata IDI-IRCCS, Rome, Italy; ^2^ Department of Translational Medicine and Laboratorio per le Tecnologie delle Terapie Avanzate (LTTA) Centre, University of Ferrara, Ferrara, Italy; ^3^ Bioinformatics Unit, Istituto Dermopatico dell'Immacolata IDI-IRCCS, Rome, Italy; ^4^ Department of Experimental Oncology, European Institute of Oncology (IEO) IRCCS, Milano, Italy; ^5^ Department of Dermatology, Istituto Dermopatico dell'Immacolata IDI-IRCCS, Rome, Italy; ^6^ Dermatology Unit, Policlinico Tor Vergata, University of Tor Vergata, Rome, Italy; ^7^ Clinical and Laboratory Molecular Allergy Unit, Istituto Dermopatico dell'Immacolata IDI-IRCCS, Rome, Italy

**Keywords:** cutaneous T-cell lymphoma, Sezary syndrome, RNA-seq, whole exome sequencing, extracorporeal photopheresis, candidates associated with therapy resistance and personalized treatment

## Abstract

**Background:**

Sézary syndrome (SS) is an aggressive and leukemic variant of Cutaneous T-cell Lymphoma (CTCL) with an incidence of 1 case per million people per year. It is characterized by a complex and heterogeneous profile of genetic alteration ns that has so far precluded the development of a specific and definitive therapeutic intervention.

**Methods:**

Deep-RNA-sequencing (RNA-seq) data were used to analyze the single nucleotide variants (SNVs) carried by 128 putative CTCL-driver genes, previously identified as mutated in genomic studies, in longitudinal SS samples collected from 17 patients subjected to extracorporeal photopheresis (ECP) with Interferon-α. Results obtained were integrated with Whole Exome Sequencing (WES) data. SNVs were validated using the Sanger method. Pathway analysis was performed with g:Profiler web server (https://biit.cs.ut.ee/gprofiler/gost). Statistical analyses were performed with GraphPad PRISM 8 software.

**Results:**

Nonsynonymous SNVs were identified in 56 genes. Integration of RNA-seq with WES data revealed that about half of these genes contained somatic mutations. Among them, the most frequently transcribed mutated genes were TET2, JAK3, NCOR1, PDCD11, RHOA, and TP53. Nearly all the remaining genes had germline-restricted mutations, and included ARID1A, ATM, ATR, CREBBP, POLD1, and POT1 genes, which are involved in DNA repair, homologous recombination, and chromatin remodeling, and the CROCC gene, implicated in centrosome cohesion. Monitoring of the mutated genes, identified within an enlarged panel of CTCL associated genes, revealed their reduction in almost 70% of SS patients as well as a significant decline of total number of mutations (SNVs) during ECP treatment. Several mutated genes persisted post-therapy, representing novel candidates associated with ECP resistance that could also have a potential prognostic relevance. Notably, these genes mainly converge on pathways related to DNA repair (ATR, ATRIP, POLD1, TP53, TP53BP1/2) which might represent novel targets to be explored in combination with ECP.

**Conclusions:**

This is the first evaluation in SS of expressed mutations in a large panel of CTCL-driver genes. Also innovative is the monitoring of mutated genes in patients’ malignant lymphocytes during ECP, a first-line treatment of CTCL, which highlights novel candidates associated with ECP resistance that might unmask novel pharmacological vulnerabilities to be exploited during ECP for a personalized treatment.

## Introduction

Cutaneous T-cell lymphoma (CTCL) is a rare tumor characterized by the expansion of malignant T lymphocytes in the skin. Among them, Sezary syndrome (SS) is the rarest but the most aggressive variant characterized, at the onset of the disease, by the co-presence of neoplastic lymphocytes, the Sezary cells, mainly in the blood, lymph-nodes and skin. Patients affected by SS have a poor prognosis with a 5-years survival as low as 24% (1).

Molecular events implicated in SS pathogenesis are multiple and heterogeneous among patients, with recurrent genetic events of gains/losses affecting mainly chromosome 8, 9, 10, and 17 (2–5). Recently, next-generation sequencing (NGS) studies showed somatic copy-number variations (SCNVs) and somatic single-nucleotide variants (SSNVs) in a broad number of genes in different pathways mainly implicated in T-cell activation and apoptosis, JAK/STAT signaling, activation of NF-κB, chromatin remodeling, and DNA damage response (6–14). Most of these studies employed whole genome (WGS) or whole exome (WES) sequencing data (6–14). However, DNA-based procedures may detect many SSNVs within exons located in the non-transcribed alleles or that have low expression, possibly representing mutations with scarce biological significance (15). An alternative strategy to identify mutations in transcribed genes that might be clinically relevant is represented by RNA sequencing (RNA-seq), commonly used for gene expression profiling (16).

We recently conducted deep RNA-seq of serial SS samples derived from 17 patients treated with extracorporeal photopheresis (ECP) in combination with Interferon-α (IFNα) to determine their gene expression profiles (*manuscript in preparation*). Taking advantage of these RNA-seq data, we performed a Variant Calling Analysis (VCA) to identify transcribed single-nucleotide variants (SNVs) in 128 putative CTCL-driver genes found mutated by previous genomic studies (6–14) and in an enlarged set of genes sharing similar domains and/or belonging to the same families or implicated in the same pathways of the 128-gene panel. Moreover, we investigated the effect of ECP treatment on tumor mutational burden and its possible prognostic value.

## Materials and methods

### Patients

This study, approved by the Ethical Committee of the Istituto Dermopatico dell’Immacolata (ID n.4/CE/2015 and n.37/CE/2023), was conducted on 35 SS samples derived from 16 SS patients with 2 serial samples and 1 patient with 3 serial samples. Three control samples were obtained from 3 Healthy Donors (HD).

The diagnosis of SS was based on the criteria described (17). Retrospective samples were obtained from patients uniformly treated with INFα (Roferon-A, 3 million IU, three times a week) and ECP performed for 2 consecutive days every month. Specifically, from SS patients 67, 78, 81, 83, 84, 85, 87, and 94 the first sample (T1) was collected before the start of therapy (baseline) or after the 1^st^ or 2^nd^ cycle of ECP, while the second sample (T2) was obtained after a mean number of ECP cycles of 19.5. For SS patient 67 an additional sample (T3) was collected after 74 ECP cycles. These patients were considered “naïve”. From SS patients 32, 45, 50, 60, 69, 76, 77, 88, and 92, T1 samples were collected after an average of 9.7 ECP cycles and T2 samples after an average of 28 ECP cycles. This second group of patients were referred as “pretreated”. More detailed patient information is available in **Supplementary Table S1**.

### Tumor cell isolation and RNA and DNA extraction

Fluorescence activated cell sorting (FACS) for clinical routine was employed to measure the absolute counts of circulating patient’s neoplastic lymphocytes (i.e. SS cells) by the detection of specific TCR-Vβ+ rearrangement (IO Test beta mark, Beckman Coulter, Fullerton, CA) in combination with anti-CD3 and anti-CD4 and/or with anti-CD3, anti-CD4, anti-CD26 and anti-CD7 monoclonal antibodies (BD Bioscience) as shown in **Supplementary Figure S1**.Circulating tumor burden, expressed as percentage of clonal CD3+CD4+TCRVβ+ and/or CD3+CD4+CD26^-^CD7^-^ cells calculated within the total CD4+ T cells for each patient is shown in **Supplementary Table S1**. Isolation of SS cells from peripheral blood (PB) was performed using the untouched human CD4+ T Cell Isolation Kit (Miltenyi Biotech, Germany) following the manufacturer instructions. Purity of samples measured by FACS assessed >90% of CD4+ T cells for each patient analyzed in this study. Matched granulocytes or CD4^-^ T lymphocytes (normal counterpart) were obtained from each patient and their purity was confirmed by FACS (% CD4^+^ <4%). RNA and DNA from SS cells and matched normal cells were isolated, quantified and evaluated for integrity as previously described (18, 19).

### RNA sequencing

TruSeq Stranded mRNA transcriptome analysis was performed on tumor (n=35) or normal (n=3) purified samples following the Illumina recommendations (Illumina, Inc). An equimolar libraries pool, measured by Bioanalyzer High Sensitivity DNA 1000 Assay and Qubit^®^ RNA HS Assay Kit, was loaded onto Illumina NextSeq 500 platform according to the manufacturer’s recommendations. To obtain a high percentage of sequencing reads, we pooled 8 library samples per run into a High Output Kit v2.5 cartridge (paired-end sequencing with about 50 million clusters per sample). Base-calling was performed by Illumina Real-Time Analysis software and NextSeq Control Software.

### Variant calling analysis from RNA-seq data

The raw RNA-seq reads from all samples were quality assessed using FastQC v. 0.11.5 a quality control tool available online at http://www.bioinformatics.babraham.ac.uk/projects/fastqc. We obtained a median of 43.5 million 74 bp paired end reads per sample. To assess the presence of variants we used the GATK Best Practices workflow for SNP and indel calling on RNA-seq data. Briefly, after the alignment, reads were split into exon segments and hard-clipped to remove any sequence overhanging into the intronic region. A base quality score recalibration algorithm adjusted the base quality scores of each base call in the data. Single Nucleotide Variants (SNVs) were called using the HaplotypeCaller tool (Calling Variants in RNA-seq: Methods and Workflows. https://www.broadinstitute.org/gatk/guide/article?id=3891). Variants were filtered by excluding clusters of at least 3 SNPs within a window of 35 bases (using the parameters -window 35 -cluster 3) and based on quality metrics, removing those with Fisher Strand values (FS) > 30.0, which indicate a high probability of strand bias at the site, and Qual By Depth values (QD) < 2.0. Resulting variants were subsequently filtered to retain only those with a Variant Allele Frequency (VAF) greater than 15% and a sequencing depth higher than 20x. To identify pathogenic variations, mutations also occurring in healthy donors’ samples were filtered out.

### Whole exome sequencing

WES libraries were generated using Twist comprehensive exome kit (Twist biosciences) according to the manufacturer’s protocol. Briefly, 50 ng of gDNA was enzymatically fragmented and adaptor sequences were added to the ends. The fragmented DNA was amplified by PCR followed by purification. Target regions were captured with Twist Comprehensive Exome Panel probes followed by PCR amplification and purification of the enriched library. Quantification of enriched libraries was performed with Qubit dsDNA High Sensitivity quantification assay kit (Thermo Fisher Scientific) and library size distribution was measured with Bioanalyzer 2100 and High Sensitivity DNA Kit (Agilent Technologies). Final DNA libraries sequencing was performed in Illumina NovaSeq 6000 platform using the S4 Reagent Kit 300 cycles (2 x 150 paired-end reads) (Illumina).

WES data were mapped against hg38 genome using DRAGMAP aligner (DRAGEN Illumina). SNV and Indel variant calling was performed with Illumina DRAGEN Bio-IT Platform v4.0 using proprietary pipelines.

After variant calling, data were collected in maf file. For downstream analyses, we filtered germline variants with a minimum coverage (DP) of 30x and variant allele frequency (VAF) higher than 20%. For somatic variants, we set our filters to 100x DP an 5% of VAF. Data were aggregated using Rstudio software embedded with maftools library. (maftool ref 10.1101/gr.239244.118).

The matched normal samples were sequenced to achieve a mean target coverage of approximately 50x. Somatic variant calling was performed using the Illumina DRAGEN Somatic Pipeline (v4.2) in tumor-normal mode with default parameters, including a VAF threshold of 0.2% in the normal sample to exclude potential germline variants.

DRAGEN employs an internal probabilistic model that integrates tumor and normal read data, base quality, mapping quality, allele frequency, and background noise estimation to identify somatic variants. The pipeline applies multiple default filters, including minimum supporting reads, variant allele frequency thresholds in both tumor and normal samples, and quality score cutoffs. It also accounts for known problematic regions through a statistical noise model, rather than fixed exclusion lists.

All filtering steps were performed using the default settings provided by the DRAGEN pipeline, which are designed to ensure high-confidence somatic mutation calls. Complete details of DRAGEN’s variant calling and filtering logic can be found in the official documentation here: https://supportdocs.illumina.com/SW/dragen_v42/Content/SW/DRAGEN/GPipelineSomCom_appDRAG.htm.

### Sanger sequencing

Genomic PCR products were subjected to direct Sanger nucleotide sequencing. Primers’ sequences are reported in **Supplementary Table S2**. The sequenced PCR products were analyzed by Chromas Lite software (Technelysium Pty Ltd, South Brisbane, Australia).

### Over-representation analysis

Biological pathways over-representation was performed with g:Profiler web server (https://biit.cs.ut.ee/gprofiler/gost) utilizing g:GOst with Reactome as data source and Benjamini-Hochberg FDR for multiple testing correction.

### Statistical analysis

Statistical analyses were performed with GraphPad PRISM 6 software (GraphPad Software Inc., La Jolla, CA). Differences were evaluated with paired/unpaired two-tailed Student’s t test, correlation test by simple linear regression and Kaplan-Meier (KM) estimator by Log-rank test/Log-rank test for trend. p≤ 0.05 was considered significant. (Mantel-Haenszel) statistical approach in survival analyses was used to estimate the Hazard Ratios (HR) with 95% confidence intervals (95% CIs) and class risk at various F-UP intervals.

## Results

### Identification of SNVs in SS by RNA-seq and WES: a comparison with literature

Several studies that have analyzed the genome of SS cells have reported several mutations. However, an extensive exploration of transcribed and thus potentially disease-relevant mutations has not yet been conducted.

With this purpose, we performed a VCA using RNA-seq data of 35 serial SS samples derived from 17 patients under ECP and INFα treatment and of 3 samples from HD used as control. As a first step, we evaluated the *global* mutational spectrum of our sample set that revealed that 50% of SNVs were synonymous substitutions while the remaining were missense (44%), frameshift (5%) and stop-gain (1%) mutations (**Supplementary Figure S2**).

**Figure 1:**
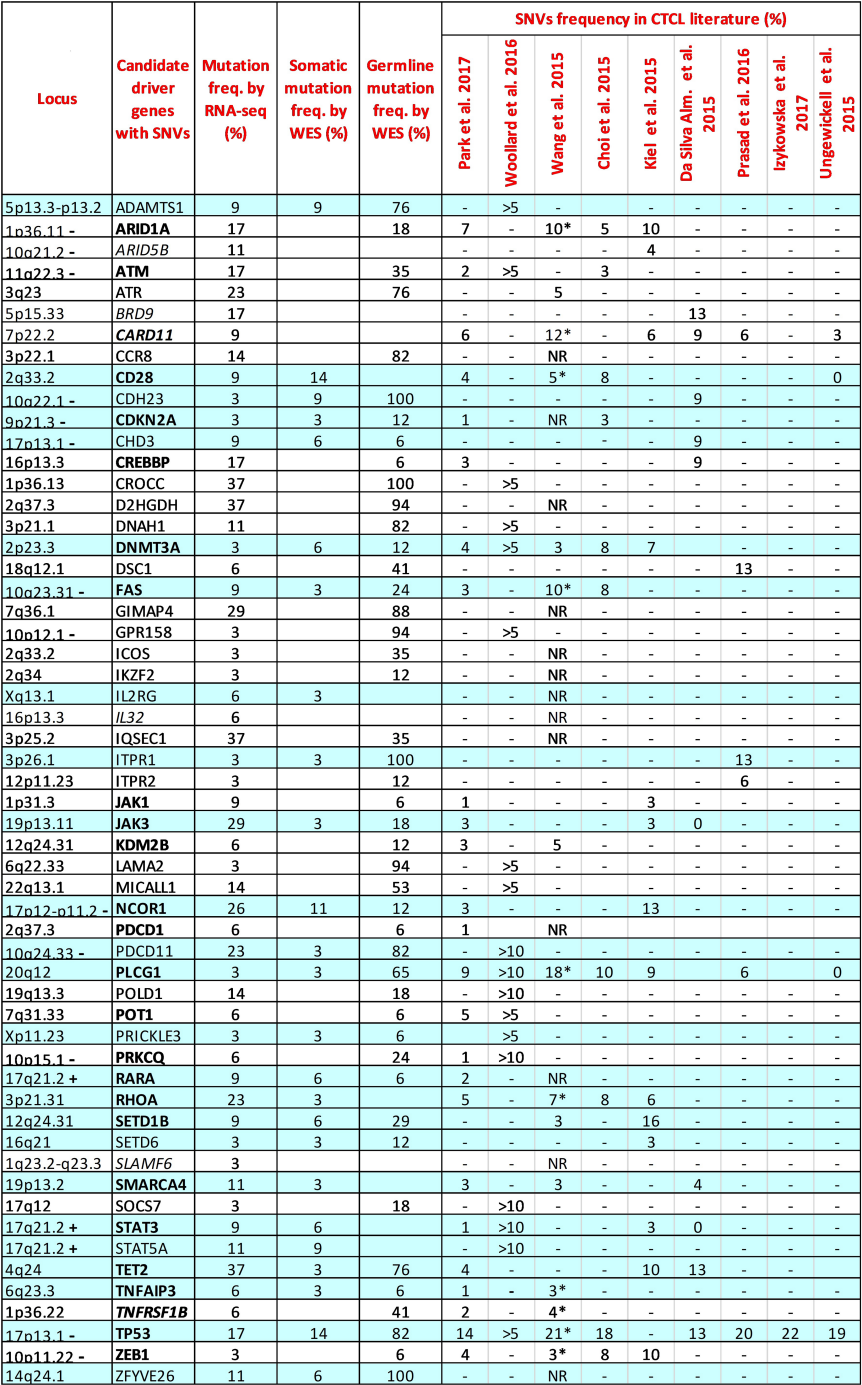
Comparison between the mutation frequencies of 56 CTCL driver genes resulting from our RNA-seq and WES data and those reported in the literature. Genes common in at least two authors are highlighted in bold. Genes without mutations detected by our WES are highlighted in italics. RNA-seq samples: n=35; WES somatic samples: n=35; WES germline samples: n=17. The reported frequencies for CTCL literature refer to the Sezary samples of each study. >5; >10: Frequency reported in more than 5% or 10% of samples investigated. *: Extension cohort (N=68 patients); NR: Frequency not reported; -: chromosome region frequently lost in SS; +: chromosome region frequently gained in SS.

We next focused our VCA on 128 candidate CTCL-driver genes identified as mutated in nine independent studies conducted by WGS or WES (6–14) (**Supplementary Table S3**). To implement the strength of our data, we filtered for nonsynonymous SNVs with coverage>20 and excluded those occurring in HD samples. As a result, 56 of the selected 128 genes were found mutated in our sample set (**Figure 1**). Among these genes, 24 (42%) showed a low mutation frequency, ranging from 3% to 6% of the samples, results that, except for a few genes, agree with the genomic data from literature (**Figure 1**).

For the 56 mutated genes, we also analyzed WES data derived from the same SS samples and paired normal counterparts. This approach allowed us to compare the mutation frequency detected by RNA-seq and WES, and to distinguish genes bearing variants with somatic or/and germline origin for the first time in detail. As shown in **Figure 1**, 25 of the 56 genes analyzed by WES had a somatic mutation frequency, obtained by filtering out germline variants, consistent with those observed in the nine reference studies (**Figure 1**, sky-blue lines). Considering the mutation frequencies obtained from the RNA-seq data for this group of genes, we also observed that JAK3, NCOR1, PDCD11, RHOA, TET2 and TP53, all strongly implicated in SS, had a higher mutation frequency (≥17% of the samples), a finding that reflects both the presence of their unfiltered germline variants and, more importantly, their effective transcription in SS cells.

WES analyses performed on paired tumor-normal samples then identified 26 genes (46.4%) carrying only mutations of germline origin (**Figure 1**, white lines). Among them, ARID1A, ATM, ATR, POLD1 and POT1 genes, all involved in genome maintenance and DNA repair (20), showed, with exception of POT1, a high frequency of mutation according to RNA-seq (ranging from 14% to 23%), suggesting a transcriptional active role in SS cells potentially favoring the onset of this lymphoma. Noteworthy, ARID1A at 1p36.11 and ATM at 11q22.3 both map on significant narrow chromosome focal deletions in SS (6), a finding that further underlines the pathogenic role of these two genes in this lymphoma.

Within the genes carrying only germline-derived mutations we also found the CROCC, D2HGDH, GIMAP4, and IQSEC1 genes, rarely reported previously and never studied in detail in SS. These genes resulted to be the most mutated by RNA-seq, with a frequency ranging from 29% to 37% of the samples, suggesting their involvement in SS pathobiology.

For the remaining ARID5B, BRD9, CARD11, IL32 and SLAMF6 genes, we were unable to detect somatic or germline mutations by WES. (**Figure 1**, in italic). This finding can be explained by the incomplete overlap between the SNVs called by the two techniques, as already demonstrated elsewhere (15).

In general, it’s interesting to note that 16 of the 56 genes analyzed (28%) map on chromosomal regions frequently lost or gained in SS (2, 3), **Figure 1**, suggesting that biallelic damage could contribute to the etiology of the disease in patients carrying mutations in these genes.

### Validation of SNVs affecting CROCC and other genes

**Figure 2:**
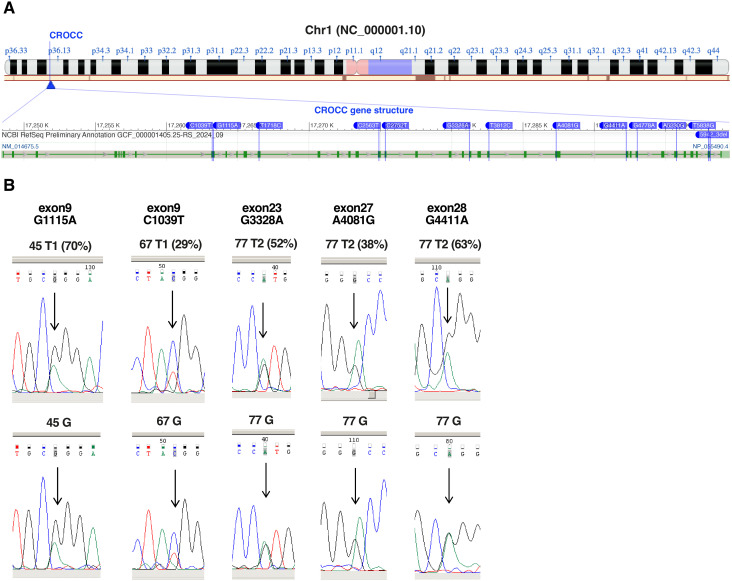
Validation of CROCC SNVs identified by RNA-Seq. **(A)** Chromosome localization (1p36.13) and gene structure of CROCC showing SNVs detected by RNA-seq (modified from NCBI)**. (B)** Chromatograms showing the sequencing of the nucleotides surrounding the highlighted SNVs (relative peak indicated by the arrow) in CROCC gene. Sequencing of the tumor sample (top, frequencies of the SNVs according to RNA-seq in brackets) and of the matched normal cells represented by granulocytes or CD4^-^ T-cells (bottom), whose purity was confirmed by cytofluorimetric analysis (% CD4^+^ <4%). G, germline.

The CROCC gene, mapping at 1p36.13 locus, resulted one of the most frequently mutated genes at germline level in our SS samples as assessed by RNA-seq and WES data. Specifically, 13 different SNVs were detected in 17 SS samples obtained from 9 patients (**Figure 2A**; **Table 1**). Importantly, all SNVs identified for this gene, except for one, were mutated more often than expected by chance (gnomAD score <0.05), which is consistent with the rarity of the disease (**Table 1**).

**Table 1:** CROCC SNVs in SS patients.

SS ID	Chr.	Position	Ref.	Alt.	ExonicFunc.refGene	AA Change. refGene	dbSNP	IMPACT	CADD	SIFT	PolyPhen	WES	Sanger
45T1	1	17263290	G	A	nonsynonymous_SNV	NM 014675:exon9:c.G1115A:p.R372Q	rs57442576	Moderate	12.47	T	B	G	G+S
60T2	1	17266498	T	C	nonsynonymous_SNV	NM_ 014675:exon13:c.T1718C:p.L573P	–	Moderate	24.9	D	Pr	–	n.a.
67T1/T3	1	17263214	C	T	nonsynonymous_SNV	NM_ 014675:exon9:c.C1039T:p.R347W	rs145088791	Moderate	23.6	D	Po	G	G+S
69T2	1	17298116	CAG	C	frameshift_deletion	NM_ 014675:exon36:c.5942_5943del: p.Q1981 fs	–	High	–	–	–	–	n.a.
77T1/T2	1	17295764	A	G	nonsynonymous_SNV	NM_014675:exon32:c.A5230G:p.S1744G	rs56278097	Moderate	19.83	T	Po	G	n.a.
77T2	1	17287301	A	G	nonsynonymous_SNV	NM_014675:exon27:c.A4081G:p.T1361A	rs76576326	Moderate	10.4	T	B	G	G+S
77T2	1	17292223	G	A	nonsynonymous_SNV	NM_014675:exon28:c.G4411A:p.G1471R	rs78888579	Moderate	16.1	T	B	G	G+S
77T2	1	17275337	C	T	nonsynonymous_SNV	NM_014675:exon19:c.C2752T:p.R918W	rs143866013	Moderate	29	D	Pr	G	n.a.
77T2	1	17281235	G	A	nonsynonymous_SNV	NM_014675:exon23:c.G3328A:p.V1110M	rs41272737	Moderate	15.5	T	B	G	G+S
78T1	1	17298013	T	G	nonsynonymous_SNV	NM_014675:exon36:c.T5838G:p.D1946E	–	Moderate	0.001	T	B	–	n.a.
83T1	1	17282599	T	C	nonsynonymous_SNV	NM_014675:exon25:c.T3812C:p.V1271A	–	Moderate	23	D	B	–	n.a.
88T1/T2	1	17274874	C	T	nonsynonymous_SNV	NM_014675:exon18:c.C2563T:p.R855W	rs200026680	Moderate	25.2	D	Po	G	n.a.
94T1/T2	1	17292984	G	A	nonsynonymous_SNV	NM_014675:exon30:c.G4778A:p.R1593Q	rs763364549	Moderate	28.4	D	Po	G	n.a.

B, Benign; T, Tolerated; Po, Possibly Damaging; Pr, Probably Damaging; D, Damaging; G, germline; S: somatic.

- = not present.

Nine of the 13 mutations were confirmed to be of germline origin by WES (**Table 1**). Notably, two mutations, namely R855W and R1593Q are predicted to be deleterious by the SIFT and Polyphen algorithms (21, 22) (**Supplementary Table S4**).

Since CROCC gene is implicated in centrosome cohesion and disjunction (23), and its mutation could play a pathogenic role in SS, we decided to further validate the mutations detected by RNA-seq data also by Sanger sequencing of patient tumor and matched normal cells. Sufficient amount of DNA extracted from purified SS cells was available only for patients 45 and 67 (T1 samples), patient 77 (T2 sample), The sequenced PCR products confirmed the presence of a benign missense R372Q (G1115A, exon 9) in T1 sample of patient 45, a deleterious missense R347W (C1039T, exon 9) in T1 sample of patient 67 and three benign missense mutation in T2 sample of patient 77, namely: T1361A (A4081G, exon 27), G1471R (G4411A, exon 28) and V1110M (G3328A, exon 23). All five mutations were present at germline level (**Figure 2B**).

To further confirm the robustness of our RNA-seq data, we also validated, by Sanger, a mutation in DH2GDH, which turns out to be another of the most mutated genes as well as a mutation occurring in TP53 for the crucial oncogenic role it plays in tumors. As shown in **Supplementary Figure S3**, we validated both at somatic and germline level, the missense mutation Y266C (A797G, exon 8) for the DH2GDH gene occurring in patient 60 (T1 sample) and the missense mutation F302C (T905G, exon 6) for TP53 gene occurring in patient 87 (T1 sample). Note that this latter mutation was not detected by WES, indicating that RNA-seq and WES methods, are not completely overlapping as already reported (15) (**Supplementary Table S4**). Conversely, WES confirmed the missense mutation Y266C for the DH2GDH gene (**Supplementary Table S4**).

WES also confirmed two deleterious missense mutations detected by RNA-seq, namely the Q61X (C181T) mutation for the TP53 gene detected at somatic level in patient 84, as well as the P626S (C1876T) mutation for the IQSEC gene at germline level of patients 60, 81, 84, 85 and 88. WES also detected this mutation in patient 67 while RNA-seq did not (**Supplementary Table S4**).

### ECP therapy reduces the mutational load in SS

We next investigated whether ECP can affect the tumor mutational burden in SS cells. To reinforce our analysis, the initial list of 128 candidate CTLC-driver genes was manually extended to include genes sharing similar domains and/or belonging to the same families or implicated in the same pathways. We identified 104 additional genes carrying non-synonymous SNVs according to RNA-seq data. The frequency of SNVs in those genes and in the 56 gene previously identified (**Figure 1**) is reported in **Supplementary Table S5**.

Notably, among these 160 genes, those mutated with a percentage ranging from 23% to 57% of samples resulted involved in: DNA damage response (ATR), centrosome assembly (CROCC and TP53BP1), epigenetic regulation (ATRX, NCOR1 and TET2), ribosomal RNA processing (HEATR1), focal adhesion and motility (IQSEC1 and RHOA), mitochondrial enzyme activity (D2HGDH) and cell homeostasis (TMEM160, TMEM131).

We then, monitored the number of mutated genes within the 160-gene panel for each patient’s samples (T1 and T2), with the only exception of patient 76 (without mutation at T1) and the T3 sample of patient 67 (total patients: n.16; total samples: n. 32).

**Figure 3:**
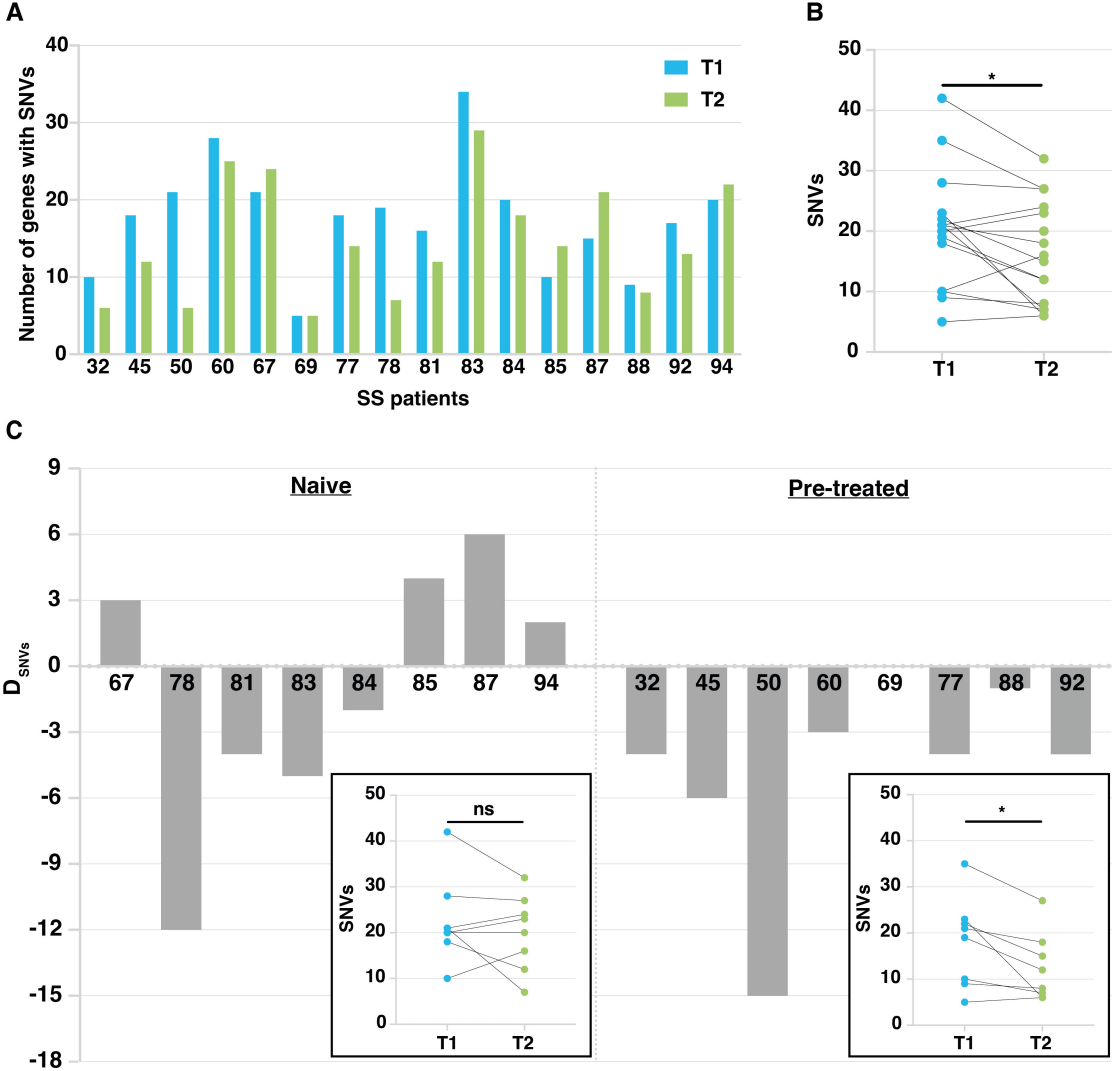
Mutation tracking in patient’s malignant lymphocytes during ECP treatment. **(A)** Histograms showing the number of mutated genes in T1 and T2 samples calculated on the total number of patients studied (n=16). **(B)** Dot plot showing mutation number (SNVs) at T1 and T2. *p<0.05 according to Paired Student’s t-test. **(C)** Histogram plots related to naïve and pretreated patients showing the difference in the number of genes affected by SNVs between T1 and T2. Patient number is on the x axis. D_SNVS =_ difference in the number of mutated genes between T1 and T2 samples. In the boxes, dot plots showing SNV number at T1 and T2 detected in naïve and pretreated patients, respectively. *p<0.05 according to Paired Student’s t-test.

We observed a reduction of the total number of mutated genes in most of SS patients (11 out of 16 patients) during the ECP treatment, with a mean of 19.1 and 13.6 mutated genes in T1 and T2 samples, respectively (**Figure 3A**). A significant decline was also observed when we compared the total number of mutations (SNVs) observed at T1 with those observed at T2(*p*<0.05) (**Figure 3B**).

. We then compared the effect of ECP on tumor mutational burden of naïve patients (n=8) assessed at T1 (baseline or after the 1^st^ or 2^nd^ ECP treatment), and at T2 (after a mean number of 19.5 ECP cycles) with that of pretreated patients (n=8) assessed at T1 (after a mean number of 8.5 ECP cycles) and T2 (after a mean number of 24.9 ECP cycles) (**Supplementary Table S1**).

Fixing an arbitrary cut-off of ±3 genes with SNVs as difference between T2 and T1 (D_SNVs_), 3 out of 8 naïve patients showed a small increase in the number of mutated genes (D_SNVs_ between 3 and 6), one patient (94) did not achieve the fixed threshold whereas the remaining four patients showed a reduction of mutated genes that appeared very marked for patient 78, showing a D_SNVs_ of -12 genes (**Figure 3C**, left). As shown, the overall SNV reduction found at T2 *vs* T1 did not reach statistical significance.

Conversely, all pretreated patients, except for patients 69 and 88 not reaching the fixed threshold, showed a reduction of mutational load. Indeed, patients 32, 45, 60, 77 and 92 showed a D_SNVs_ between -3 and -6 genes, whereas patient 50 presented a noticeable D_SNVs_ of -15 genes (**Figure 3C**, right). Overall, a significant decline of the number of SNVs found at T2 *vs* T1 was observed (*p*<0.05).Taken together, these results indicate that: 1) increasing the number of ECP cycles decreases the mutational load significantly; 2) nevertheless, a substantial proportion of mutated genes identified in T1 samples, persists after therapy (T2 samples) in both naïve and pretreated patients.

### Persistent mutated genes after ECP have a potential prognostic relevance

**Figure 4:**
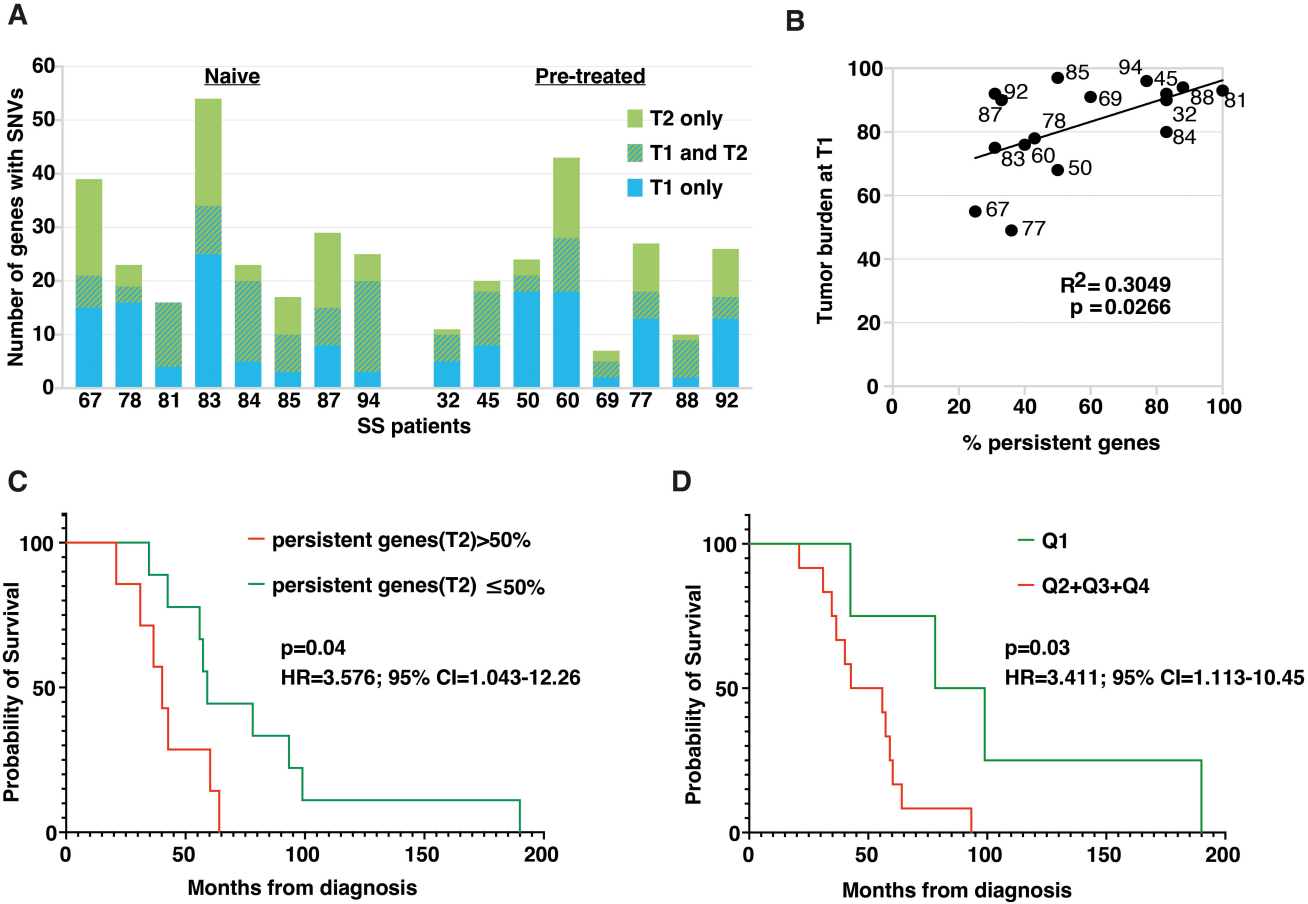
Persistent and common genes emerging after ECP treatment have a prognostic relevance for SS patients. **(A)** Histogram plot showing the number of genes affected by SNVs at T1 and T2. Hatched bars represent persistent mutated genes, i.e. genes that present SNVs at both T1 and T2. **(B)** Dot plot showing the positive Pearson’s correlation between tumor burden at T1, expressed as percentage of clonal CD4+TCRVβ+ T-cells calculated within total CD4+ T-cells, and percentage of persistent mutated genes calculated on total mutated genes at T2 for each individual. **(C)** Kaplan-Meier analysis comparing OS between patients bearing more than 50% of persistent mutated genes at T2 (red, n=7) and patients for whom the percentage was less than or equal to 50% (green, n=9). Significance was calculated by the log-rank test. Patients with >50% of mutations showed an increased risk of death (HR: 3.57. 95% CI: 1.043-12.26). **(D)** Kaplan-Meier analysis comparing survival between patients with <20.25% of 15-commonly mutated genes at T2 (1^st^ quartile) and all other patients with ≥20.25% of mutations at T2 (2^nd^-4^th^ quartile). Significance was calculated by the log-rank test. Patients with ≥20.25% of mutations showed an increased risk of death (HR: 3.4. 95% CI: 1.13-10.45).

The presence of mutated genes persisting in T2 samples of SS patients undergoing ECP could represent an index of resistance to treatment and assume a prognostic significance. We thus looked at genes carrying SNVs that were present in both T1 and T2 samples derived from all patients analyzed (naïve and pretreated). Results revealed a heterogeneous percentage of persistent mutated genes in both groups, with a mean percentage of 53% ± 27% for naïve and of 43% ± 21% for pretreated patients (**Figure 4A**).

We then wondered if there was any relationship between tumor burden, evaluated as number of circulating neoplastic cells at T1, expressed as percentage of clonal CD4+TCRVβ+ calculated within total CD4+ T cells, and the number of persistent mutated genes found in each patient. Results obtained revealed a significant positive correlation between these two variables (n=16, r^2^ = 0.3049, p=0.0266), indicating that as tumor burden increases, the percentage of persistent genes found after ECP also increases (**Figure 4B**).

We also asked if an association existed between patients’ overall survival (OS) and the percentage of persistent mutated genes. To this end, patients were clustered into two groups based on the median values (i.e. 50%) of the percentage of persistent mutated genes calculated for everyone.

KM survival analysis revealed that patients with more than 50% of persistent mutated genes at T2 had a worse prognosis (median OS 42.3 ± 15.4 months, n=7) compared to patients whose percentage of persistent mutated genes was ≤50% (median OS 78.9 ± 46.9, n=9) (*P*=0.04 for Log-rank test) (**Figure 4C**). and had an increased risk of death (HR: 3.57. 95% CI: 1.043-12.26) also shown by the risk table **Supplementary Table S6A**.

Finally, to identify the mutated genes that most frequently persisted after ECP, we focused on those found in both T1 and T2 samples in at least three patients. Applying this rule, we highlighted 15 commonly persistent mutated genes (carrying the same SNV or different SNVs) that are listed in **Table 2**. As shown, almost all mutations were also detected by WES, confirming the reliability of our RNA-seq data (**Table 2**).

**Table 2:** Integration of RNA seq and WES data for fifteen persistent mutated genes commonly found in patients after ECP.

Locus	Gene	HGVS	Impact	CADD (PHRED)	SIFT	Poly Phen	Effect	Patients															Total
								32	45	60	67	69	77	78	81	83	84	85	87	88	92	94	
chr3: 142178144	**ATR**	NM_001184:exon43:c.G7274A:p.R2425Q	M	7.66	T	B	missense								•		•	•					3
chr2: 27439751	**ATRAID**	NM_001170795:exon7:c.G625T:p.A209S	M	18.01	T	B	missense			•	•						•						3
chr1: 17274874	**CROCC**	NM_014675:exon18:c.C2563T:p.R855W	M	25.2	D	Po	missense													•			1
chr1: 17292984	**CROCC**	NM_014675:exon30:c.G4778A:p.R1593Q	M	28.4	D	Po	missense															•	1
chr1: 17295764	**CROCC**	NM_014675:exon32:c.A5230G:p.S1744G	M	19.83	T	Po	missense						•										1
chr2: 242690745	**D2HGDH**	NM_001287249:exon7:c.C680T:p.A227V	M	0.002	T	B	missense						•		•		•					•	4
chr2: 242695322	**D2HGDH**	NM_001287249:exon8:c.A797G:p.Y266C	M	24.4	D	Po	missense			•													1
chr2: 242695394	**D2HGDH**	NM_001287249:exon8:c.C869T:p.P290L	M	12.50	T	B	missense					•											1
chr7: 150269542	**GIMAP4**	NM_018326:exon3:c.G384T:p.E128D	M	13.03	T	B	missense	•		•			•	•							•		5
chr1: 236729956	**HEATR1**	NM_018072:exon30:c.A4298G:p.Y1433C	M	2.78	T	B	missense		•									•					2
chr1: 236723108	**HEATR1**	NM_018072:exon34:c.G4676A:p.S1559N	M	8.55	T	B	missense										•						1
chr3: 12962074	**IQSEC1**	NM_001134382:exon6:c.C1876T:p.P626S	M	23.80	D	Po	missense			•					•		•	•		•			5
chr19: 17950294	**JAK3**	NM_000215:exon10:c.G1433A:p.R478K	M	16.30	T	B	missense									•							1
chr19: 17945696	**JAK3**	NM_000215:exon16:c.G2164A:p.V722I	M	8.63	T	B	missense	•													•		2
chr19: 17942142	**JAK3**	NM_000215:exon21:c.A2873C:p.E958A	M	23.0	T	B	missense										•						1
chr4: 106196951	**TET2**	NM_001127208:exon11:c.A5284G:p.I1762V	M	0.07	T	B	missense		•						•			•		•		•	5
chr9: 35712003	**TLN1**	NM_006289:exon28:c.C3680T:p.S1227L	M	22.90	T	B	missense		•									•				•	3
chr11: 102272884	**TMEM123**	NM_052932:exon3:c.G211A:p.V71M	M	8.36	T	B	missense										•		•				2
chr11: 102272839	**TMEM123**	NM_052932:exon3:c.G256T:p.V86F	M	10.85	D	B	missense							•									1
chr2: 98388811	**TMEM131**	NM_015348:exon33:c.4396dupA:p.S1466fs	H	.	.	.	frameshift, insertion															•	1
chr2: 98373842	**TMEM131**	NM_015348:exon41:c.T5372A:p.V1791D	M	23.80	D(LC)	B	missense									•			•				2
chr19: 47549454	**TMEM160**	NM_017854:exon3:c.G358A:p.G120S	M	11.76	T	B	missense			•	•	•	•			•	•		•		•	•	9
chr1: 202976622	**TMEM183A**	NM_001079809:exon1:c.G29T:p.R10M	M	21.70	T (LC)	B	missense								•								1
chr1: 202977809	**TMEM183A**	NM_001079809:exon3:c.G238A:p.A80T	M	19.67	T	B	missense			•	•										•		3
chr17: 7579389	**TP53**	NM_001126118:exon3:c.C181T:p.Q61X	H	34.0	.	.	stop gain										•						1
chr17: 7574018	**TP53**	NM_001126118:exon6:c.C892T:p.R298C	M	20.7	T	B	missense									•							1
chr17: 7574005	**TP53**	NM_001126118:exon6:c.T905G:p.F302C	M	29.0	D	Po	missense												•				1
							Total	2	3	6	3	2	4	2	5	4	9	5	4	3	4	6	62

RNAseq data are represented by dots; WES data are indicated by green box (germline) and by yellow box (somatic).

HGVS, Human Genome Variation Society Database; Impact: M, moderate; H, high; The CADD-SV scores on the PHRED scale range from 0 (potentially benign) to 48 (potentially pathogenic);

SIFT: D, deleterious; T, tolerated; Poly Phen: B, benign; LC, low confidence; Po, probably damaging; Total refers only to SNVs identified by RNAseq.

Then, we asked whether the presence of persistent mutated genes belonging to this more restricted panel might also have prognostic significance. To this end, patients were stratified into quartiles according to the percentage of persistent mutated genes belonging to the 15-gene panel (Q1 = 20.25%; Q2 = 29%; Q3 = 37.5%; Q4 = 50%). KM survival analysis (**Figure 4D**) revealed that patients with <20.25% of these mutated genes at T2 (1^st^ quartile) had the best prognosis compared with all other patients with ≥ 20.25% at T2. (2^nd^- 4^th^ quartile) *(*p=0.03 for log rank test.). An increased risk of death (HR: 3.4. 95% CI: 1.13-10.45) was also evidenced for this latter group of patients and the relative risk is shown in **Supplementary Table S6B**.

### Analysis of pathways affected by mutated genes detected in patients under ECP treatment

**Figure 5:**
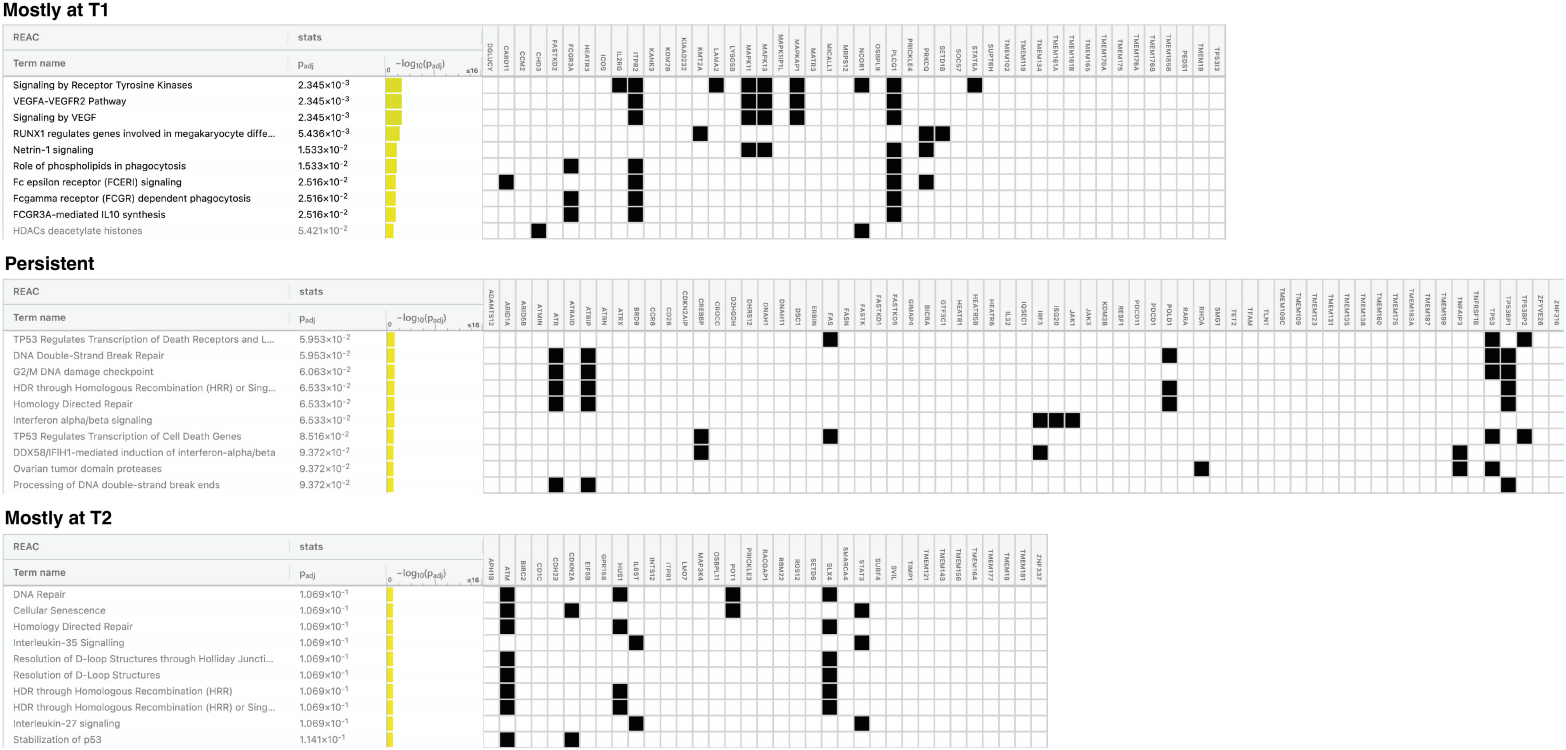
Pathways most affected by mutated genes found in naïve and pretreated patients during the disease course. Figure showing the top 10 pathways for each gene list queried: genes mutated mostly at T1, at both T1 and T2 (persistent) and mostly at T2. Pathways in bold are those significantly over-represented. Padj = False Discovery Rate (FDR).

To understand which biological effects could be influenced most by the mutated genes within the 160-gene panel, we used g:Profiler to perform over-representation analysis of Reactome pathways. To perform this analysis, we queried the Reactome with the lists of genes found mutated mainly at T1 (n=46), T1 and T2 (n=67) or mainly at T2 samples (n=35) retrieved considering the totality of patients (**Supplementary Table S7**; **Figure 5**). Our manually extended gene set was set as custom statistical domain. Note that 12 genes were excluded from this survey because they were found with the same frequency in either T1 or T2 samples (**Supplementary Table S7**).

Considering the genes mutated mostly in T1 samples, we detected a significant over-representation of pathways mainly related to tyrosin kinase receptor and VEGF signaling, RUNX1 activity and Netrin1 signaling. (Padj ≤2.5x10^-2^).

For the persistent genes, an over-representation approaching the statistical significance was observed for TP53-mediated transcriptional regulation of death genes (receptors, ligands and effectors), DNA double-strand break repair, G2/M DNA damage checkpoint, Homology-Directed Repair (HDR) and INFα/β signaling pathways (Padj ≤ 6.5x 10^-2^) The genes restricted to the T2 samples resulted mainly implicated in DNA repair, cellular senescence, HDR, IL-35/27 signaling and resolution of D-loop structures pathways, without reaching statistical significance.

Similar, though not entirely overlapping, results were obtained when the Reactome analysis was restricted to the naïve group (**Supplementary Figure S4**). The main divergence concerns the HDR and DNA damage repair (DDR) signaling pathways mainly found over-represented in T2-associated genes (Padj 4.8x10^-2^) and not within persistent genes as seen in the total of patients, indicating that these pathways are engaged after exposure to ECP. Exclusive of the naïve-T2 samples appears Notch signaling. Notably, over-representation of all pathways identified in T2-associtated genes were significant, possibly reflecting a greater homogeneity of naive samples.

Finally, for the 15 persistent genes most frequently mutated we did not find any over-represented functional pathway, a result that may be due to the limited number of genes analyzed.

## Discussion

SS presents a complex and highly heterogeneous genomic landscape, with diverse driver gene mutations identified by several NGS studies (24, 25). However, the relevance of these mutations for SS biological behavior and response or resistance to therapy remains largely unclear. Since the biological significance of genetic changes depends on whether the mutated genes are expressed or not, here we used RNA-seq data of 35 longitudinal SS cell samples obtained from 17 patients treated with ECP and IFNα to identify transcribed SNVs in 128 CTCL-driver genes found mutated by previous genomic studies (6–14) and more recent investigations (26, 27). Among these, we identified 56 genes carrying mutations altering the amino-acid sequence of the encoded protein (nonsynonymous SNVs).

WES analyses of tumor-normal matched samples revealed that, after filtering out the germline SNVs, 25 of these 56 genes, were affected by somatic mutations while nearly all the remaining genes had only germline-derived mutations. Considering the RNA-seq data, we observed that 40% of the 56 mutated genes had a low mutation frequency, suggesting that they might have little relevance to the SS pathobiology or that these genes might be less transcribed due to the reported mutations.

Among the genes harboring somatic mutations, TET2, JAK3, NCOR1, PDCD11, RHOA and TP53 showed the highest rate of mutations (≥17%) according to RNA-seq data, a result reflecting their implication in the pathogenesis of SS as already described (28).

Within the genes carrying a high rate of only germline-derived mutations we observed ATM and ATR, involved in DNA-damage responses, POLD1, which participates in homologous recombination and ARID1A and CREBBP, both involved in chromatin remodeling (29). Notably, germline-derived alterations in ATM, ATR and POLD1 genes could amplify the TP53 mutations detected in our samples and therefore contribute to the known genomic instability of SS (28). Similarly, germline mutations in ARID1A and CREBBP could contribute to epigenetic defects arising from alterations in the NCOR1, TET2, and DNMT3A genes, implicated in histone acetylation and DNA methylation mechanisms, reported here and by others (30).

Within genes harboring only germline-derived mutations, we also found a subset of novel or poorly studied genes, such as CROCC, D2HGDH, GIMAP4, and IQSEC1, showing the highest mutation rate (≥29%). Notably, CROCC gene, which codes for an important component of the ciliary root, a structure involved in centrosome cohesion prior to mitosis (23), could play a pathogenic role in SS similarly to what has been observed in colorectal cancer (31). This hypothesis finds support in the quality of CROCC mutations detected here since at least two of them result deleterious according to SIFT and Polyphen algorithms. It should be noted that centrosome abnormalities are frequently found in both solid and hematologic cancers and can cause a failure of spindle assembly, resulting in unbalanced segregation of chromosomes during mitosis and genomic instability (23), a characteristic of SS (9).

Overall, the results of RNA-seq and WES conducted in our set of samples not only identify mutated genes that are effectively transcribed, but also highlights an abundance of germline mutations that, occurring at the exome level might affect the function of the encoded proteins thus promoting tumorigenesis. However, functional studies such as cell-based assays, gene overexpression or knockdown, or CRISPR gene editing are needed to determine the actual biological significance or pathogenic role in SS of the mutations identified here and, therefore, their clinical relevance.

Although new therapies and treatment combinations have recently emerged (32), ECP remains a safe and well tolerated first-line treatment for aggressive CTCLs (33, 34). It is based on the collection of patients’ leukocytes that are first treated with a photoactivatable drug, the 8-methoxypsoralen (8-MOP), and then exposed to ultraviolet A (UVA) light radiation before being reinfused into patients (33, 34). Recently, a phase I/II clinical trial, involving ECP in combination with 5-aminolevulinic acid, a drug that is more selective in targeting neoplastic T cells than 8-MOP, has also been conducted in patients with CTCL (*ClinicalTrials.gov ID NCT03109353*), proving to be safe and well-tolerate (35). The mechanism of action of ECP is still under investigation however it is well established that ECP treatment induces apoptosis of CTCL neoplastic cells, normalize the imbalance of Th1/Th2 cytokine profile and increases the number and the activation of NK and T-reg cells (36). A recent study demonstrated that normal lymphocytes exposed to ECP show an increased number of DNA double-strand breaks accompanied by enhanced expression of γ-H2AX and TP53BP1, which are biomarkers of DNA repair (37). In addition to genotoxicity, DNA-damaging therapy can stimulate anticancer immune-responses by inducing the expression of INF through the Stimulator of Interferon Genes (STING) pathway, which is activated when DNA is detected in the cytoplasm after chemotherapy and radiotherapy (38, 39). It is noteworthy that the expression of type III INF via STING pathway is induced in CTCL cells exposed to 8-MOP and UVA, indicating that this circuit is activated also by ECP (40).

ECP leads to prolonged disease control (41) and recent studies have investigated hematological parameters (42) and cytokines (36) as predictive biomarkers of response to therapy. However, the mechanisms underlying the efficacy of ECP and tumor cell acquired resistance to this therapy need to be further investigated (43). To shed light on these mechanisms, we monitored the number of mutated genes in SS cells during ECP therapy. We found that the mutational load was reduced by ECP, particularly in patients who had received a greater number of ECP cycles. We also observed that a lower initial tumor burden was correlated with a lower rate of persistent mutations after therapy. This finding is consistent with two large retrospective cohort studies (41, 44), showing that ECP is particularly effective when administered early, i.e. in patients with a low tumor burden, and as a first-line treatment, i.e. when an appreciable healthy immune counterpart is still present in the patients.

Although ECP produces a reduction of the mutation load, a consistent number of mutated genes continue to be present after therapy, suggesting that these could represent candidates associated with ECP resistance. Importantly, persistent mutated genes showed a prognostic relevance, as evidenced by the shorter survival and the increased risk of death of patients carrying a higher percentage of them within all those detected post-therapy. These findings, while promising, should be however interpreted with caution and confirmed in a larger cohort of patients.

Interestingly, patients with worse prognosis showed a mutational profile post ECP (T2) that was largely shared with that observed at the beginning of therapy (T1), suggesting the presence of clonal cells basically unaffected by treatment. In contrast, patients with a better prognosis displayed a more heterogeneous and broader mutational profile after therapy, suggesting the appearance of new, probably less aggressive sub-clones, under ECP pressure, as previously documented at the single-cell level in SS after histone deacetylase inhibitor (HDACi) treatment employed alone or in combination with ECP (45, 46).

Reactome analysis allowed us to identify the pathways most affected by the mutated genes here identified. In T1 samples we detected an overrepresentation of pathways involving signaling by tyrosine kinases, VEGF, RUNX1 and Netrin1, indicating an active interaction between neoplastic cells and tumor microenvironment (TME). These signals appear disused during subsequent ECP cycles possibly due to the elimination of SS cell sub-clones. Alternatively, TME changes caused by ECP, such as the Th2-Th1 cytokine profile shift (36), may preclude the crosstalk between TME and SS cells leading to transcriptional changes. This hypothesis is consistent with the expression profile changes found in SS cells after ECP exposure (47).

Persistent mutated genes result mainly implicated in TP53 transcriptional regulation of death genes (receptors, ligands, cytochrome release) in agreement with the up-regulation of TP53 and FAS/FAS ligand observed in SS following ECP exposure (47). An enrichment of mutated genes involved in DNA-double strand break repair, G2/M DNA damage checkpoint and HDR pathways were also detected, suggesting that the mutations/dysfunction of TP53 and DNA repair pathways can promote resistance of malignant cells to ECP-induced DNA damage.

Mutated genes restricted to T2 samples are implicated in DNA repair and senescence, a stress-inducible state of terminal cell cycle arrest which had been previously observed in ECP-treated cells (48, 49). Mutations in genes involved ILs signaling and in resolution of D-loop structures emerging during DNA repair synthesis were also detected suggesting cell attempt to repair the DNA damage caused by ECP treatment.

Altered DNA damage responses (DDRs) represent an opportunity to fight cancer. Indeed, defective DDRs influence both the anti-tumor immune-response by activating the INF pathway (40) and the immune-surveillance, through the accumulation of neo-antigens induced by expressed somatic mutations (38, 39) that can enhance the efficacy of immune-checkpoint (IC) inhibitors, therapeutics also used to treat SS (50).

DNA repair and DNA proofreading activity are the two main mechanisms to ensure the fidelity of DNA replication. In this scenario, mutation of POLD1 gene, which has a DNA-proofreading function, is strongly correlated with high mutation load and neo-antigen generation, thus representing a potential marker for predicting the efficacy of IC inhibitor therapy in different types of cancer (51), including SS (50). Thus, it is interesting to point out that we found that the POLD1 gene carries germline-restricted mutations with a high frequency in our sample-set.

Cells use different pathways to repair distinct forms of DNA damage, and, in general, tumors that lack specific DDR pathways often depend on other intact DDR pathways for survival, thus unmasking new therapeutic targets through the principle of synthetic lethality (52). Novel compounds against DDR pathways, already suggested for the treatment of hematological cancers (52), including SS (9, 28), might be specifically used in combination with ECP. This approach might represent a novel therapeutic strategy to treat SS, in line with the increased sensitivity to phototherapy observed in CTCL cell lines concurrently treated with small molecules against ATR signaling (53). Notably, a phase 1 clinical study involving the ATR kinase inhibitor ceralasertib used alone or in combination with radio-therapy (54) (*ClinicalTrials.gov ID NCT02223923*) has been conducted in advanced solid tumors based on the hypothesis that tumors lacking important DNA repair functions can be responsive to ceralasertib, accordingly to the principle of synthetic lethality, and that the drug may increase the efficacy of radiotherapy by preventing repair of DNA damage. The recently published results of the study (55) have indeed shown that ceralasertib monotherapy was associated with durable responses in tumors with defects in DDR and, interestingly in ARID1A that we found frequently mutated at germline level in our SS samples.

Although our study provides novel information about SS mutational landscape and suggests future treatment options for this lymphoma, it has some limitations that need to be considered. It is a retrospective investigation that, nevertheless, included a fair number of samples, given the rarity of the disease. Furthermore, studies are required to functionally characterize the mutations found in our cohort of patients. Pre-clinical studies are also needed to verify the value of the potential therapeutic targets highlighted here.

To our knowledge, this is the first evaluation in SS of expressed mutations in a large panel of putative CTCL-driver genes. Integration of RNA-seq and WES data revealed an abundance of genes with mutations of exclusive germline inheritance that could potentially represent risk factors for the development of this lymphoma, such as those affecting the CROCC gene. Future functional studies are, however, required to validate this hypothesis.

Another novel feature of the present study is the monitoring of mutated genes in patients’ malignant lymphocytes during ECP treatment. Emerging findings highlight new candidates associated with ECP resistance that converge mainly on DNA repair pathways which could increase the load of tumor neoantigens potentially enhancing ECP-mediated anti-clonal immunity in SS. Our results also suggest that therapies targeting DNA repair pathway could be exploited in combination with ECP for the treatment of SS.

## Data Availability

The RNA-seq raw data generated and analyzed during the current study are available in the NCBI Gene Expression Omnibus (GEO) data repository with the accession number GSE302772. WES tumor vs normal data are available in the European Variation Archive (EVA) with accession number PRJEB94533. In compliance with GDPR, genomic raw data will be made available to qualified researchers upon reasonable request and appropriate ethical approvals.
