# Supplementary material for: Expressed mutated genes in Sezary syndrome and their potential prognostic value in patients treated with extracorporeal photopheresis
Source: Front Immunol. 2025 Aug 22;16:1589467. doi: 10.3389/fimmu.2025.1589467 (PMC12411188; doi:10.3389/fimmu.2025.1589467)
Supplement: Supplementary file 1 [file DataSheet1.docx]

Supplementary Material

Supplementary Table S1. **Clinical features of SS patients analyzed by RNA-seq and WES**

Supplementary Table S2. **Sequence of PCR primers for variant validation and germline analysis**


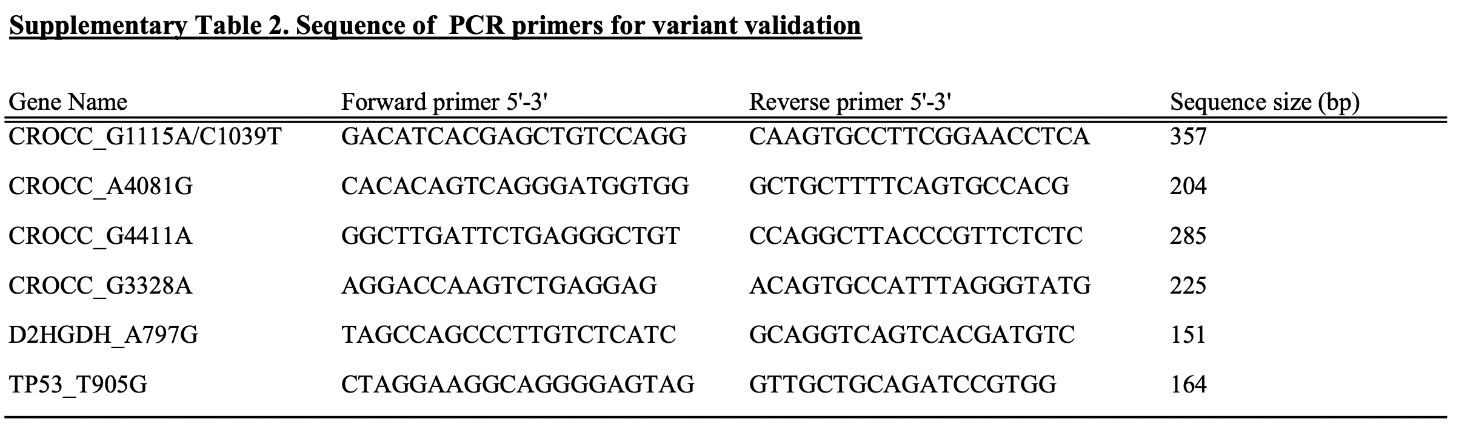


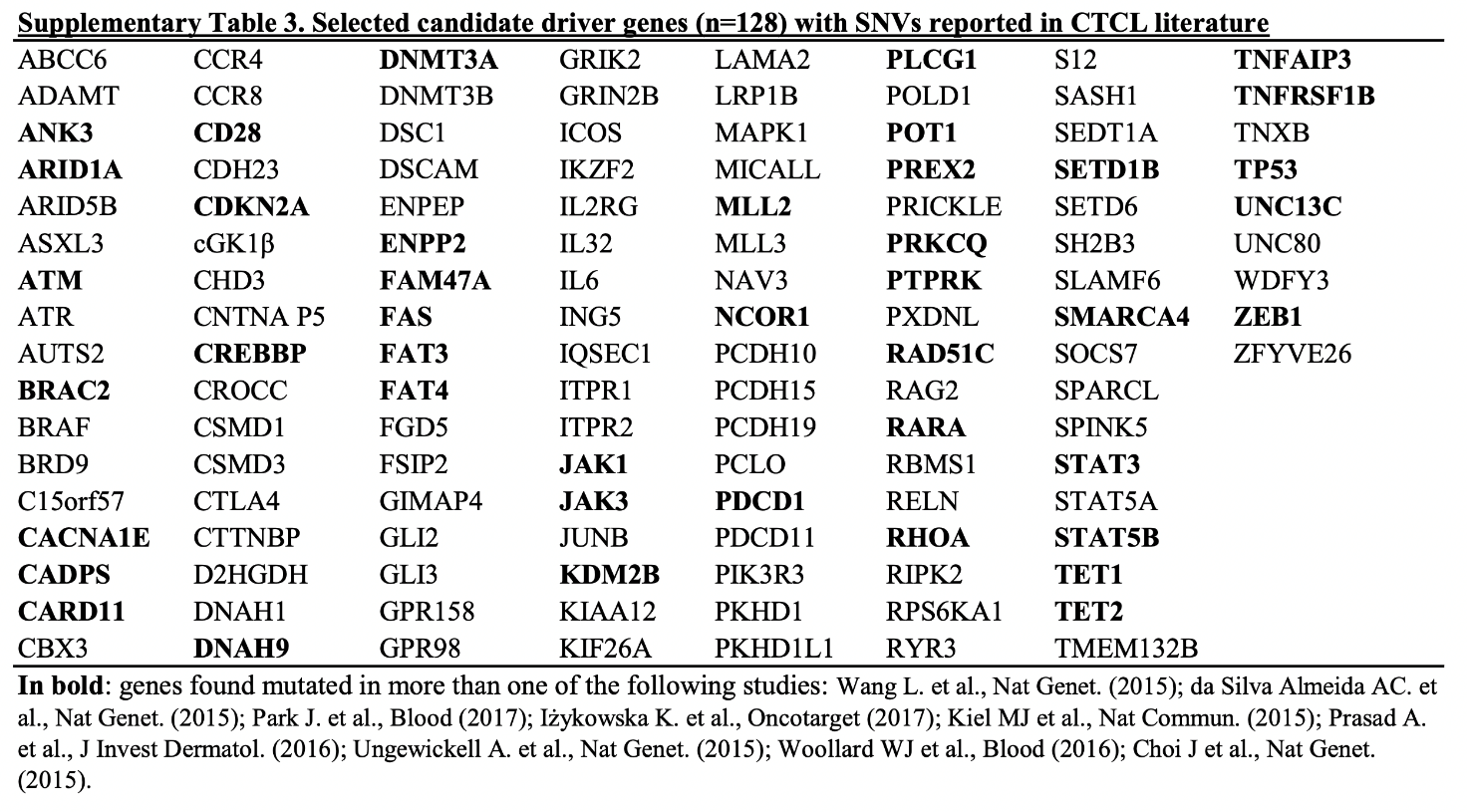
Supplementary Table S3: **Selected candidate CTCL-driver genes (n=128) with SSNVs reported by literature**

Supplementary Table S4. **Integrated analysis by WES data of deleterious mutations in four selected genes** RNAseq data (represented by dots) were integrated with germline (green box) and somatic (yellow box) WES data.

Supplementary Table S5. **List of 160 genes with SNVs identified in our SS samples and their corresponding frequencies according to RNA-seq.** Genes highlighted in blue are the 104 identified after manually extending the initial list of 128 candidate CTLC driver genes to include genes sharing similar domains and/or belonging to the same families or implicated in the same pathways


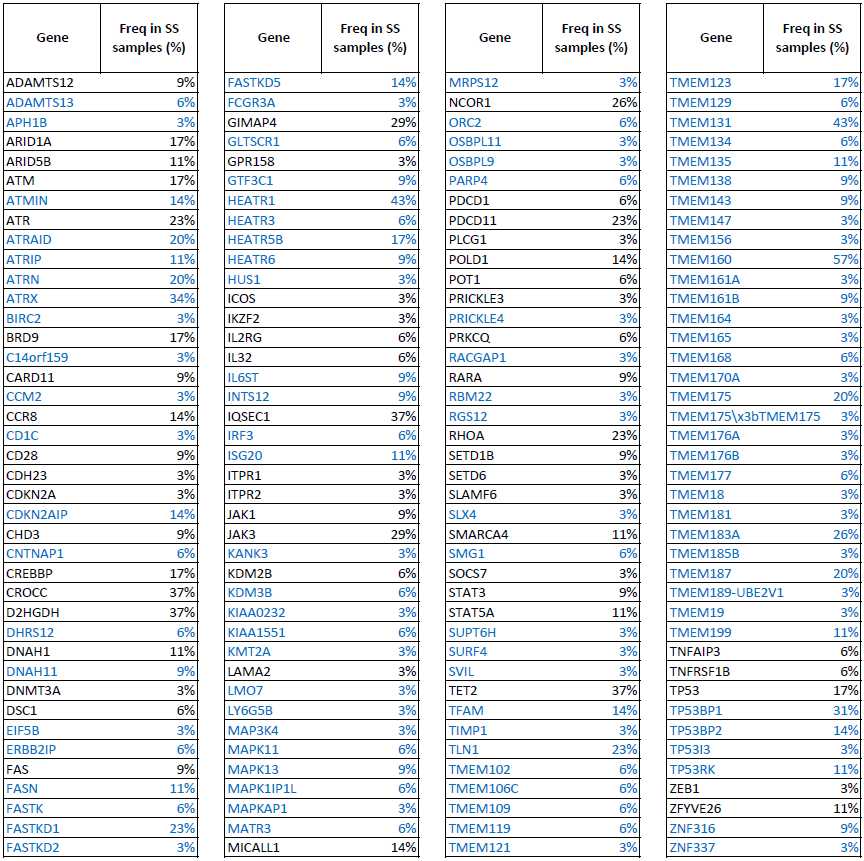


Supplementary Table S6. **Risk prediction analysis based on percentage of mutated genes at T2 and survival time (months)**. **A)** analysis performed on patients stratified based on the median values (i.e. 50%) of the percentage of persistent mutated genes calculated at T2 for everyone. **B)** analysis performed on patients stratified into quartiles according to the percentage of persistent mutated genes belonging to the 15-gene panel (Q1=20.25%; Q2=29%; Q3=37.5%; Q4=50%)


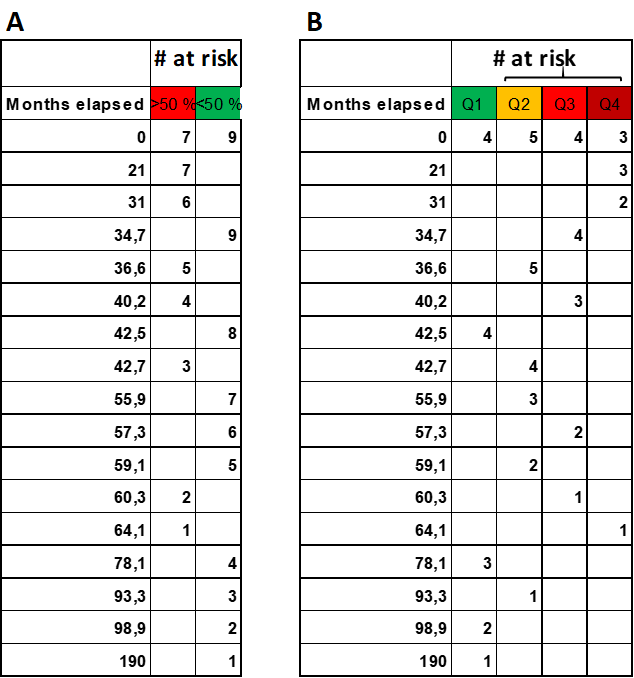


Supplementary Table S7. **List of genes for reactome analysis.**


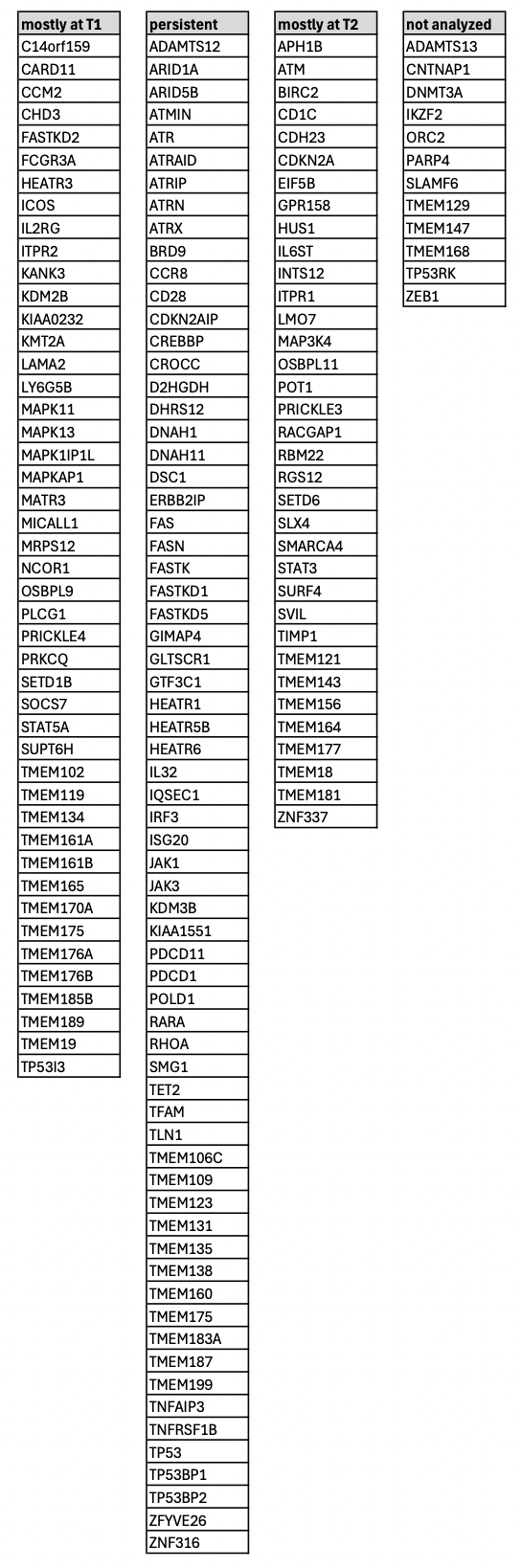


Supplementary Figure S1. **Circulating tumor burden assessed by FACS.** Upper panel: PBMCs collected at baseline of one representative patient (SS83) were co-stained with anti-TCR-Vβ 13.1 (mix-D) and anti-CD4 mAbs. Percentage of SS cells was evaluated in pre-gated CD4+ T cells as showed into the plots. Lower panel: patient’s PBMCs were also co-stained with anti-CD3 anti-CD4 anti-CD7 and anti-CD26, percentage of SS cells was calculated within total CD4+ T cells


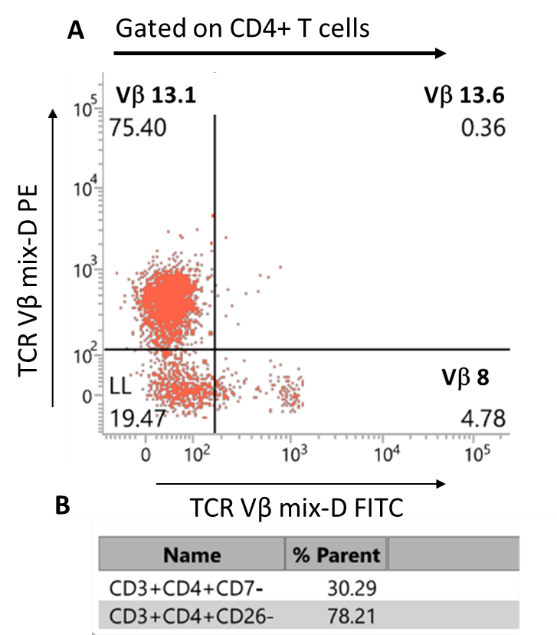


Supplementary Figure S2**.** **Histograms showing the percentages of the SNV types for each SS patient and healthy donors (HD).**


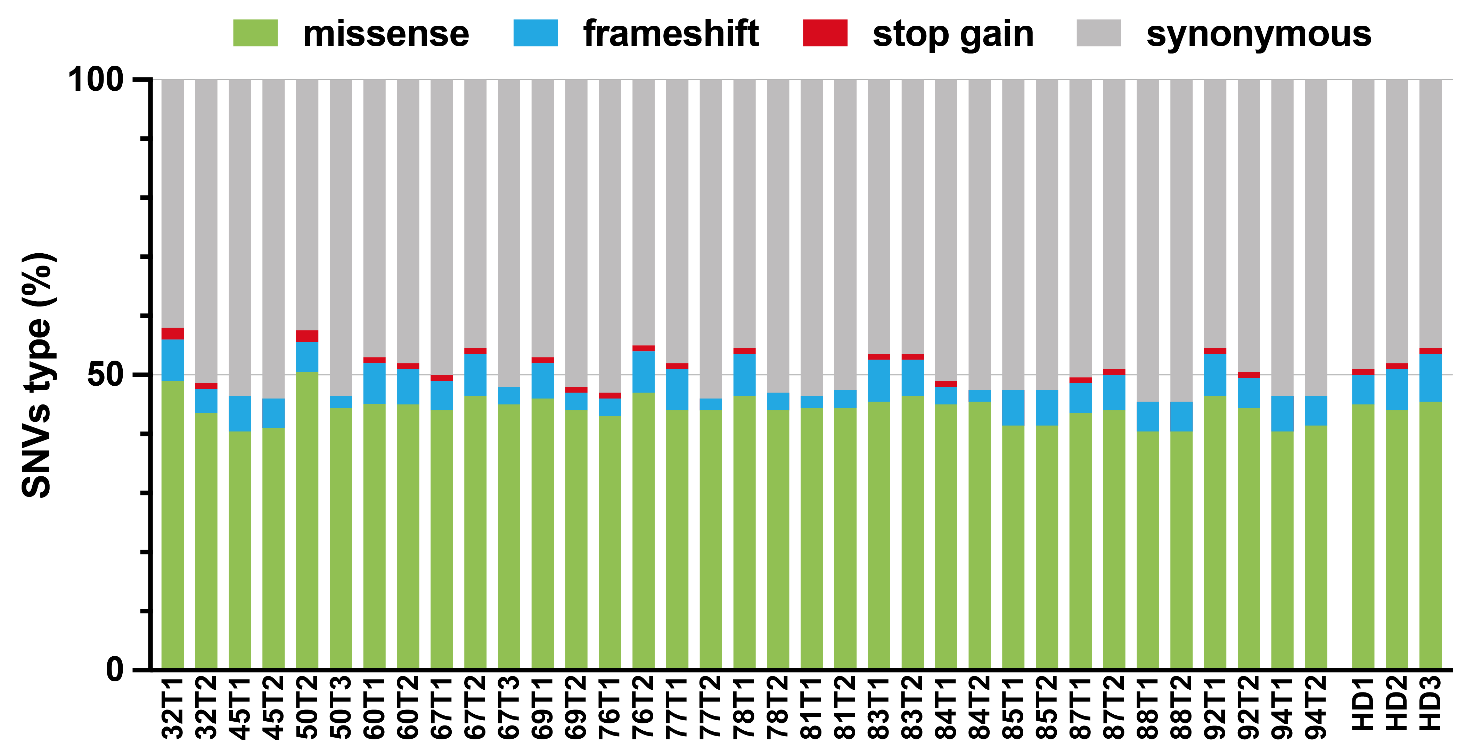


Supplementary Figure S3. **Validation of D2HGDH and TP53 SNVs identified by RNA Seq**. Chromatograms showing the sequencing of the nucleotides surrounding the highlighted SNVs (relative peak indicated by the arrow) in D2HGDH and TP53 genes. Sequencing of the tumor sample (top, frequencies of the SNVs according to RNA-seq in brackets) and of the matched normal cells (bottom), whose purity was confirmed by cytofluorimetric analysis (% CD4^+^ <4%). G = germline.


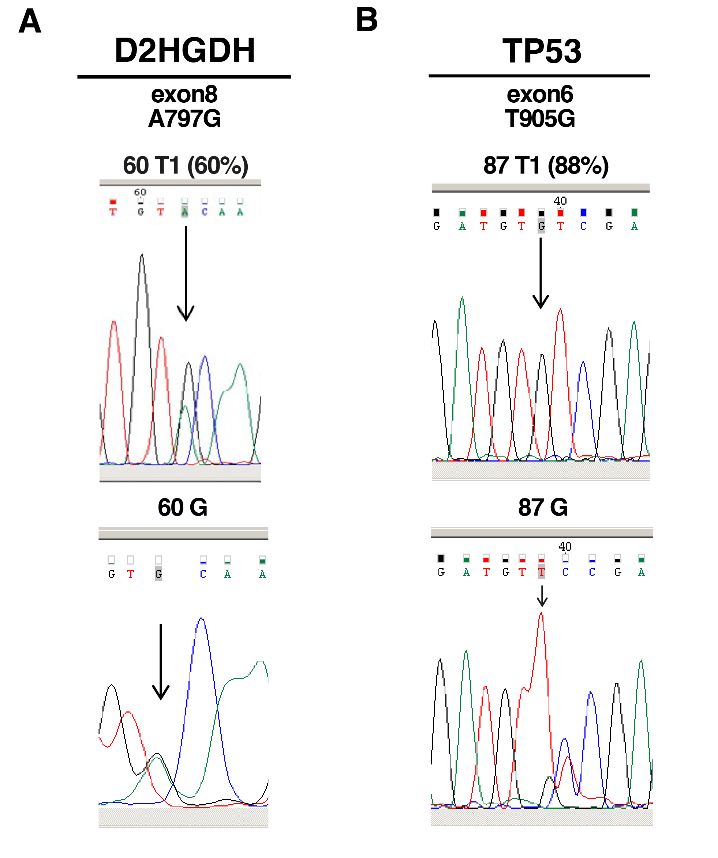


Supplementary Figure S4. **Pathways most affected by mutated genes found in naive patients during the disease course**. Tables showing top over-represented pathways (rows) for each gene list queried (columns): genes mutated mostly at T1 (top), at both T1 and T2 (center) and mostly at T2 (bottom). Pathways in black are those significantly over-represented. Padj = False Discovery Rate (FDR).

**
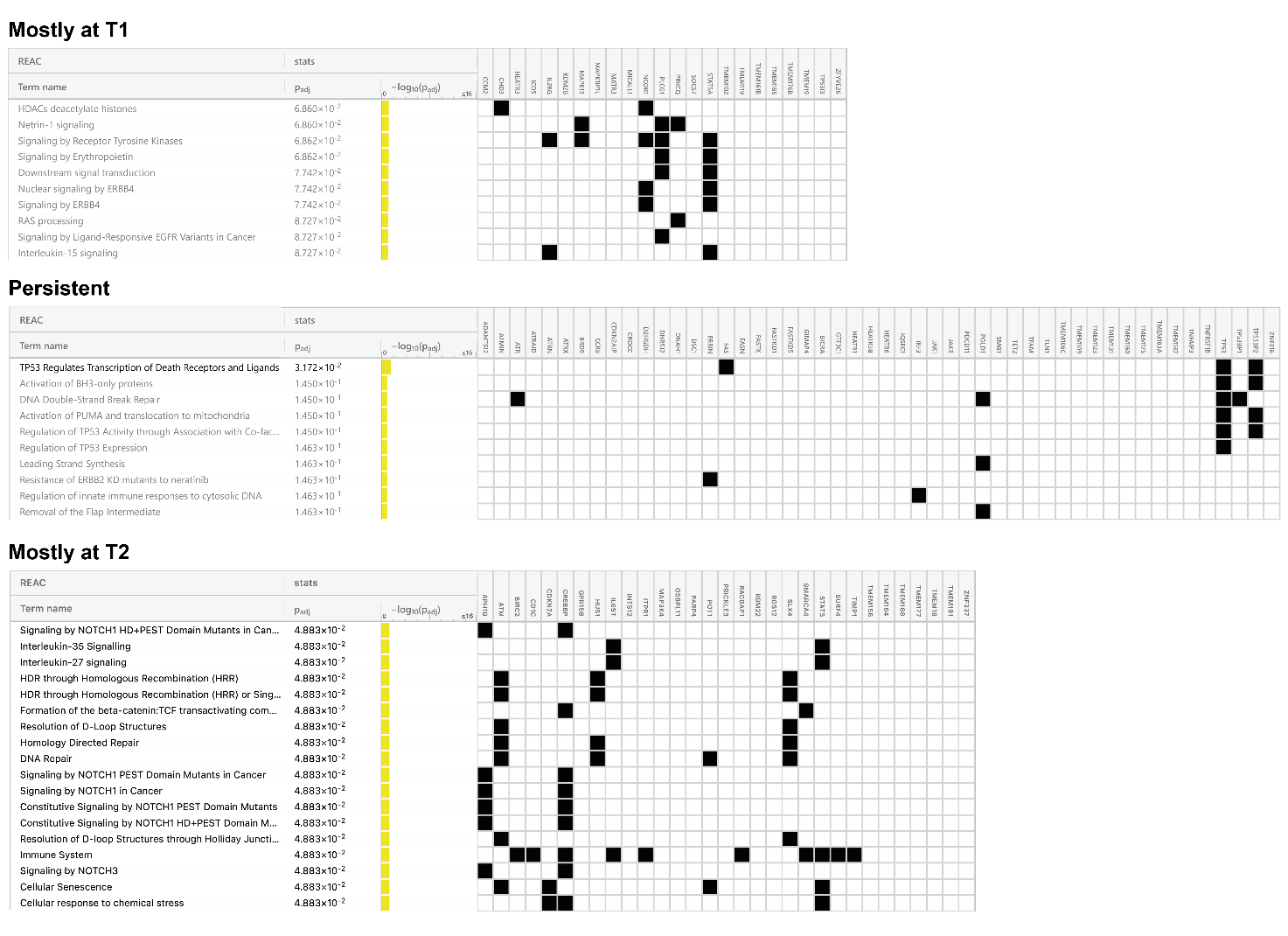
**
